# Evaluating methods to explore antibiotic use on smallholding pig farms in peri-urban Kenya

**DOI:** 10.3389/fvets.2025.1570092

**Published:** 2025-07-23

**Authors:** Claire Scott, Nicholas Bor, Kristen Klara Reyher, Alex J. Tasker, Henry Buller, Max Korir, Dishon M. Muloi, Irene Bueno, Lian Francesca Thomas

**Affiliations:** ^1^The Bristol Veterinary School, University of Bristol, Langford, North Somerset, United Kingdom; ^2^Department of Animal and Human Health, International Livestock Research Institute, Nairobi, Kenya; ^3^Department of Geography, University of Exeter, Exeter, United Kingdom; ^4^Institute of Infection, Veterinary and Ecological Sciences, University of Liverpool, Leahurst Campus, Neston, United Kingdom; ^5^Royal (Dick) School of Veterinary Studies, University of Edinburgh, Edinburgh, United Kingdom

**Keywords:** antibiotic use, evaluate, Kenya, methods, pig production, smallholder

## Abstract

**Background:**

Understanding patterns and practices of antibiotic use (ABU) in livestock is crucial to make informed recommendations for improved antibiotic stewardship and to measure the impact of interventions aimed at reducing inappropriate ABU. In the absence of a unified tool to determine ABU at the farm level, we aimed to enhance the understanding of methodological approaches used to explore ABU by evaluating the strengths and limitations of four different methods on smallholding pig farms in a peri-urban area of Nairobi, Kenya.

**Methods:**

ABU collection methods were trialed in parallel over one month on 13 farms. We evaluated four methods for their effectiveness in collecting instances of ABU and facilitating further exploration of ABU practices using qualitative discussion. The methods were: waste bucket analysis; medicine-recording sheets; weekly semi-structured interviews; and the “Drug Bag” medicine sorting technique.

**Results:**

We found that no single method captured all likely or reported instances of ABU. Waste bucket analysis collected the lowest number of instances of reported ABU. The “Drug Bag” collected the highest number of instances but risked over-reporting due to misrecognition, duplication, and recall errors. Contextual factors, such as ABU practices specific to the study context, affected methodological success. An example of this was individual animal treatments being the mainstay of antibiotic use, meaning that empty packaging was not available for the waste bucket. The use of multiple methods in parallel and qualitative data collection was helpful in ascertaining the likelihood of over- or under-reporting of ABU and allowed us to gather a more detailed understanding of ABU practices.

**Discussion:**

Our results highlight the challenges of gathering accurate farm-level ABU data. Future studies must consider methodological suitability when planning data collection; we recommend that methodological suitability statements should be included in future publications. Triangulation of methods and qualitative data collection should be employed where possible. Comparative analyses between ABU studies should be carefully structured to account for both methodological and contextual variation.

## Introduction

Estimating the quantity of livestock antibiotic use (ABU) in high-income settings with established medicine supply chains currently relies mainly on the availability of antibiotic sales data (1). Such monitoring systems rarely provide insight into decision-making about ABU at the farm level, including indications for use. In low- and middle-income countries (LMICs), antibiotics are often acquired through informal markets. Veterinary sales data may not be collected (2) or may be derived from country-level import data, which are insufficiently detailed to attribute ABU to the individual farm level (3). Currently, ABU data attributable to the individual farm level are primarily collected through one-off research projects using a variety of methods (4), which has led some authors to caution those wishing to compare these data (5), because methods to determine ABU may not be interchangeable (6, 7).

One method employed to gather farm-level ABU data has been the use of questionnaires to evaluate knowledge, awareness, and experiences of using antibiotics (8–10). These tools rely heavily on accurate recall over extended periods and across varying circumstances, presenting a very real risk of introducing recall biases and inconsistencies (11–14).

With an aim of countering these weaknesses, waste bucket or bin analysis requires participants to retain empty antibiotic packaging over a time period, allowing for a more nuanced understanding of volumes and types of antibiotics (15–17). Overall, the usefulness of waste bucket analysis to quantify ABU has been described as variable, with under-reporting possible (7, 17).

Although written treatment records are required by law in many high-income countries, studies have found ABU records to be inaccurate (18, 19) and have identified other methods (particularly waste bucket analysis) as more successful in capturing ABU (18–22). Written treatment records for ABU have rarely been utilized in studies in LMIC settings, possibly because they are not consistently legally required and may be infrequently kept by farmers. Redding et al. (7) describe how collecting written treatment records could be challenging in contexts where farmers may be illiterate.

Dixon et al. (23) suggest an alternative to understanding ABU—especially in the context of LMICs—which has since been used by others (24, 25). The “Drug Bag” is an exercise in which participants sort a bag of antibiotics brought onto the farm by researchers into sequential piles, finishing with the antibiotics used on the farm in the last month. Although they detail its limitations, Dixon et al. (23) describe the “Drug Bag” as a method that “can produce accurate ABU data as well as provide a talking point for participants to discuss antibiotic experiences” (23) (p. 1). The “Drug Bag” has not, prior to this study, been evaluated against other ABU collection methods.

Studies have highlighted concerns around conformance to antibiotic withdrawal periods for pig farms supplying a local independent abattoir (LIA) in Kiambu County, Kenya, which provides pork to the domestic market in Nairobi (26). Bor et al. (26) tested pork meat juices from a local independent abattoir found that 41% of samples (adjusted for diagnostic test performance) tested positive for antibiotic residues above EU legal limits. Previous work by Murungi et al. (27) found that brokers (those who buy pigs from farmers and sell them to traders taking pigs to abattoirs) reported that farmers often did not conform to antibiotic withdrawal periods. We, therefore, aimed to gain a deeper understanding of the factors affecting conformance to antibiotic withdrawal periods on pig farms in Kiambu County, Kenya, by identifying instances of ABU and exploring ABU practices at the farm level.

An accurate understanding of ABU at the farm level is critical to inform the design of evidence-based interventions to reduce inappropriate ABU and for evaluating the success of such interventions (3–5, 28–30). As no one method had previously been determined as most appropriate to capture farm-level ABU for our specific context, we undertook a problem identification and exploration project to evaluate methods for determining ABU in this context. We trialed four methods in parallel: waste bucket analysis; medicine-recording sheets; weekly semi-structured interviews (SSIs); and the “Drug Bag.”

Also crucial to identifying and targeting inappropriate ABU is understanding ABU practices, which describe how antibiotics are used by the end-user (31), and include the volumes, types, indications for use and so on. While several studies have provided quantitative evaluations of methods to capture ABU at the farm level (7, 19, 22), few studies provide a qualitative assessment of the utility of each method [see (18), as an exception].

In this paper, we offer an explorative, qualitative assessment of our experiences using these different techniques for capturing the nature and extent of ABU on pig farms in Kiambu County, Kenya, over one month. This was with the aim of selecting appropriate ABU collection methods for use in this context and advancing the understanding of methodological approaches used to explore ABU on farms.

## Materials and methods

The description of methods that follows can also be found in our related manuscript (32, 33). A positionality statement can also be found in this related manuscript.

### Study site and participants

We aimed to examine ABU practices on pig farms supplying pigs to a particular LIA, given the specific concerns identified by Bor et al. (26). The majority of the pigs supplied to this LIA are from local pig farms in Kiambu County, a peri-urban county bordering Nairobi, the capital city of Kenya (27, 34, 35). Therefore, we recruited 13 farms situated in one of the four sub-counties within Kiambu County that are geographically closest to the LIA and that supplied pigs to one of two LIAs situated in the county. Ten farms supplied pigs exclusively to an LIA, and three sold pigs mostly to a larger integrated processor, only selling pigs to an LIA in specific circumstances.

### Implementation

**Figure 1:**
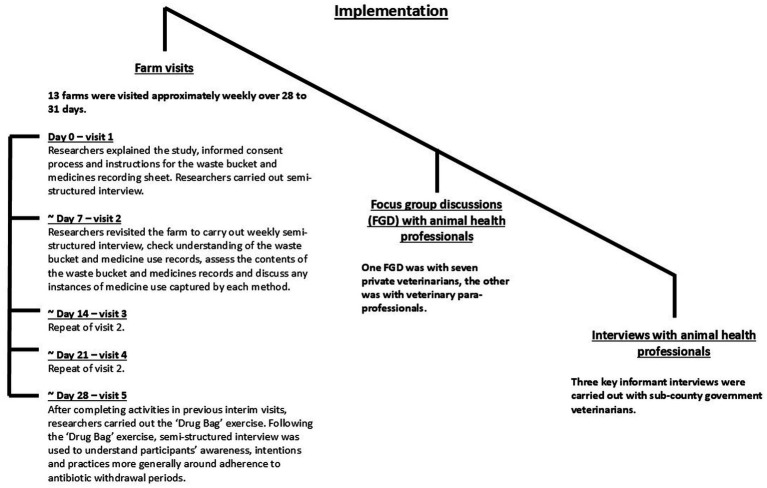
The distribution of farms and agrovets (livestock medicine shops) visited for this study, evaluating methods to explore antibiotic use on smallholding pig farms in peri-urban Kenya.

An implementation schedule is shown in Figure 1, and all interview and focus group discussion (FGD) guides can be found in Supplementary material 1.

### Farm visits

The schedule of farm visits was as per Figure 1. Farms were identified by government animal health professionals (AHPs) and asked to take part in the project. To ensure appropriate biosecurity, we disinfected boots and equipment between visits, wore disposable overalls and parked the research vehicle either off the farm or disinfected tires between visits. Upon completion of the final visit, we provided participants with protective boots, a scrubbing brush and disinfectant (approximately 12 USD in value). We also created a feedback booklet (Supplementary material 2) based on our observations throughout the project, which we gave to participants at the end of the final visit, alongside additional medicine-recording sheets for the farmer to use if they wished. Participants also kept the bucket and clipboard used for the study and were welcome to retain our disposable overalls after each visit. We interviewed participants who spoke English (Nine out of 13 farmers) in English, as this was the language spoken by the main researcher (CS). Participants who did not speak English (Four out of 13 farmers) were interviewed in Kiswahili, with Kiswahili–English translation provided by a research assistant with knowledge of the project aims and objectives (NB).

We considered two typologies of ABU evaluation methods: prospective ABU recording (waste buckets and medicine-recording sheets) and retrospective ABU recording (weekly SSI and the “Drug Bag”).

#### Prospective ABU recording

##### Waste buckets and medicine-recording sheets

**Figure 2:**
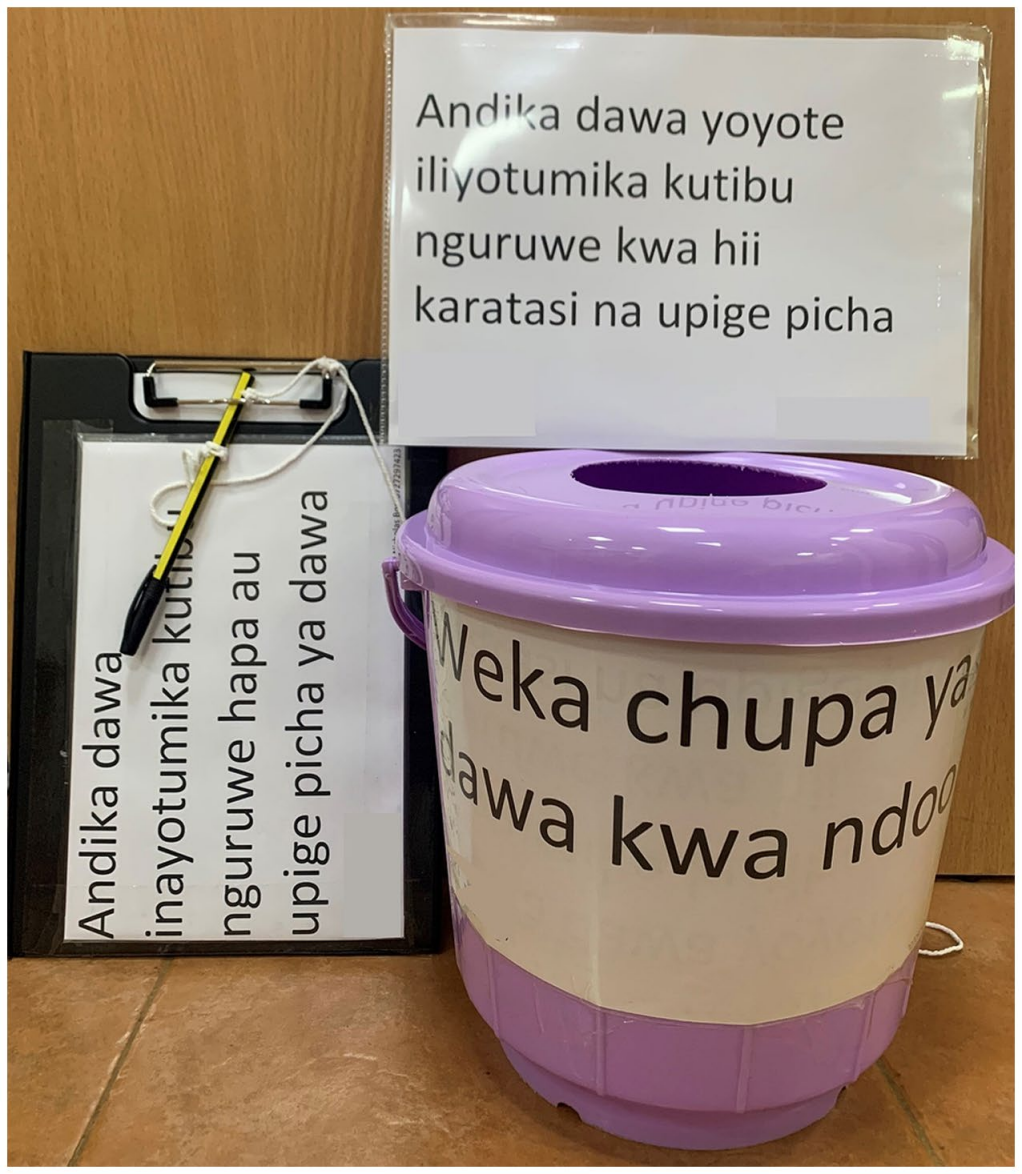
The signage waste bucket and medicine-recording sheet prototype material used to capture antibiotic use in this study, evaluating methods to explore antibiotic use on smallholding pig farms in peri-urban Kenya.

Signage, waste buckets, and medicine-recording sheets (Figure 2) were placed in a visible location on each farm. We asked farmers to use the waste bucket to deposit all packaging (bottles, sachets, etc.) of any medicines used for pigs on the farm for the month following the initial visit. We requested farmers to use the medicine-recording sheets to record any medicines used for pigs on the farm, including those administered by an AHP, for the same period. We visited participants approximately weekly for the next 28 to 31 days to confirm that the farmers had understood the instructions for both methods and to assess and discuss the contents of the waste bucket as well as any instances of medicine use recorded on the medicine-recording sheet.

#### Retrospective ABU recall

##### Weekly semi-structured interviews

At the initial visit, we conducted a farm walk and SSI with participants to explore general pig management, discuss any medicines kept on the farm and how each was used and examine the packaging of pig food to ascertain whether antibiotics were labeled for inclusion. Following the initial visit, we visited participants approximately weekly for 28 to 31 days for SSI to discuss events from the previous week relating to disease, medicine use and pig movement on and off the farm.

##### The “Drug Bag”

To prepare the “Drug Bag” (23), CS and NB visited 15 livestock medicine shops (agrovets) in the local areas surrounding the enrolled farms to purchase (or photograph, where antibiotics could not be purchased) antibiotics (Figure 1). Forty-two antibiotics were purchased (36) or photographed (6) and were numerically labeled. A table of the antibiotics included in the “Drug Bag” is provided in Supplementary material 3. To alleviate ethical challenges experienced by Dixon et al. (23), we emptied all antibiotic packaging and disposed of the medicines via incinerated before assembling the “Drug Bag.” We created signs to help participants sort the medicines into piles.

We conducted the “Drug Bag” exercise on the final visit to farms, between 28 and 31 days after the initial visit, following weekly SSI and audits and discussions regarding the waste buckets and medicine-recording sheets. As per Dixon et al. (23), we first asked participants to sort the contents of the bag into medicines they recognized and those they did not recognize. Then, we asked participants to sort the recognized medicines into those they had used for pigs and those they had not used for pigs. Participants then sorted the medicines they had used for pigs into those they had used frequently and those they had not used frequently. The final sort was then recombined into the pile of antibiotics that participants had used for pigs, and they were asked to sort this pile again, this time into the medicines they had used in the last month and those not used in the last month for pigs. We held qualitative discussions throughout the sorting to understand ABU practices. At the end of the exercise, we conducted further qualitative discussions to explore, in particular, ABU practices for the antibiotics which participants had placed in the ‘used in the last month’ pile.

### Focus group discussions and key informant interviews with animal health professionals

CS and NB conducted FGDs and key informant interviews (KIIs) with AHPs who supervise pig farms in the county to gain a greater understanding of ABU in the study context and to provide insight into the workings of our ABU collection methods, including why we may have identified strengths and limitations for each method in this context. We carried out one FGD with seven private veterinarians and another with five veterinary para-professionals. We recruited private veterinarians and veterinary para-professionals by visiting agrovet stores, through snowball sampling, social media groups, and our own contacts. We conducted three KIIs with sub-county government veterinarians, whom we recruited by contacting their office directly. As compensation for their time, we provided government veterinarians with KES 1000 (approximately 8 USD) to complete an SSI, where we visited them at work for under one hour. Private veterinarians were given KES 2000, and veterinary para-professionals were given KES 1000 to attend an FGD at the International Livestock Research Institute (ILRI) for two hours. We conducted FGDs and KIIs in English.

### Data management and analysis

CS recorded and transcribed all interviews and FGDs with the assistance of digital transcription software (36). We analyzed all transcribed data qualitatively using NVivo qualitative analysis software for thematic analysis (37), assigning the strengths and limitations of each method as deductive codes.

We photographed the contents of waste buckets and medicine-recording sheets and documented “Drug Bag” sorts using a paper recording system. We collated reported instances of ABU during the one-month study duration for each method from audio recordings, photos, and “Drug Bag” paper recording sheets. We defined one reported ABU instance as the reported use of one oral or injectable product containing antibiotics, given to one group of animals (or one individual animal) at one distinct point in time. In this way, the use of one antibiotic product containing multiple antibiotic ingredients was counted as one instance of reported ABU. Different products reportedly given at the same time were counted as separate ABU instances. Topical antibiotic products (e.g., oxytetracycline spray reported to be used after the castration of piglets) were not included.

We inputted data into a Microsoft Excel (38) spreadsheet which detailed the reported instances of ABU for each participant and for each method. We triangulated reported ABU instances captured over the study period for waste bucket analysis, medicine-recording sheets, and weekly SSI, as well as the antibiotics placed in the “used in the last month” pile for the “Drug Bag,” to understand whether methods had collected similar or different results. Where there were discrepancies between methods, we re-consulted qualitative data to try to understand why this was the case, i.e., whether reported instances of ABU were likely to constitute over- or under-reporting of ABU. To derive the themes described in our results, CS appraised the strengths and limitations of ABU collection methods together. CS generated seven themes describing recurrent ideas that she interpreted as underpinning our data.

## Results

**Figure 3:**
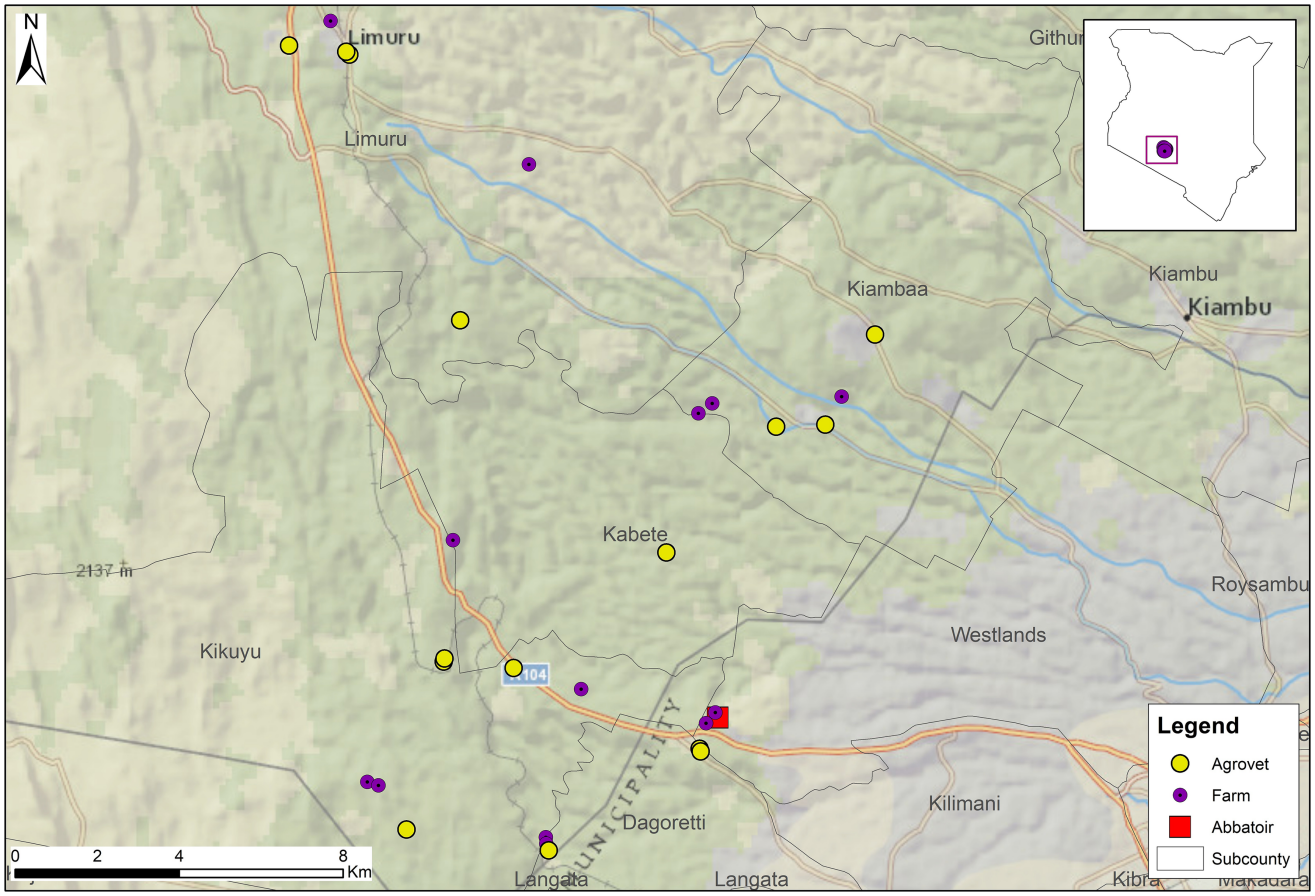
The implementation of this study, evaluating methods to explore antibiotic use on smallholding pig farms in peri-urban Kenya.

Figure 3 shows the distribution of farms recruited for the study, and Table 1 details the characteristics of farms and participating farmers. Farms ranged in size from two to 72 pigs during the initial visit; ten of the 13 farms kept fewer than 50 pigs at that time. Farms were contextually defined through a series of professional conversations with AHPs working in the county to be small (under 50 pigs) to medium-sized (50–200 pigs). All participants received an initial and final visit, ranging from 28 to 31 days apart. Twelve of the 13 participants received all interim visits; one participant (Farmer 3) declined two interim visits. Initial visits to farms lasted from ten to 50 minutes, interim visits lasted between five and 40 minutes, and final visits lasted from 20 to 45 minutes.

**Table 1:** Characteristics of farms and farmers recruited for the study alongside instances of reported ABU captured in the study.

Farm number	Farm sub-county	Participant role on the farm	Participant gender	Main outlet for pigs (LIA/LIP)	Number of pigs on first visit				Number of pigs sold for slaughter during project		Interviews conducted in:	Did the participant keep antibiotics on the farm to administer to pigs themselves?
					Total	Sows	Unweaned piglets	Other	To LIA	To LIP		
1	Kiambaa	Farm hand	Male	LIA	51	3	23	25	4	0	Kiswahili	No
2	Kiambaa	Farm hand	Female	LIA	18	1	0	17	0	0	Kiswahili	No
3	Kabete	Farm owner	Male	LIA	16	2	14	0	0	0	English	No
4	Kabete	Joint farm owner	Female	LIA	35	3	9	23	3	0	English	No
5	Kiambaa	Farm hand	Male	LIA	13	0	0	13	7	0	Kiswahili	No
6	Kabete	Farm owner	Male	LIP	74	0	0	74	0	20	English	Yes
7	Kabete	Farm owner	Male	LIP	50	2	12	38	0	12	English	Yes
8	Limuru	Farm owner	Female	LIP	40	1	9	30	0	20	English	Yes
9	Limuru	Farm manager	Male	LIA	15	0	0	15	0	0	English	Yes
10	Kikuyu	Farm owner	Female	LIA	23	1	13	9	0	0	Kiswahili	No
11	Kikuyu	Farm owner	Female	LIA	34	3	16	15	0	0	English	No
12	Kikuyu	Farm owner	Male	LIA	16	2	0	16	0	0	English	No
13	Kikuyu	Farm owner	Male	LIA	2	0	0	2	0	0	English	No
Totals					387	18	96	277	14	52		

Sows are defined as being of parity one or more. SSI, semi-structured interview; LIA, local independent abattoir; LIP, large integrated processor.

**Figure 4:**
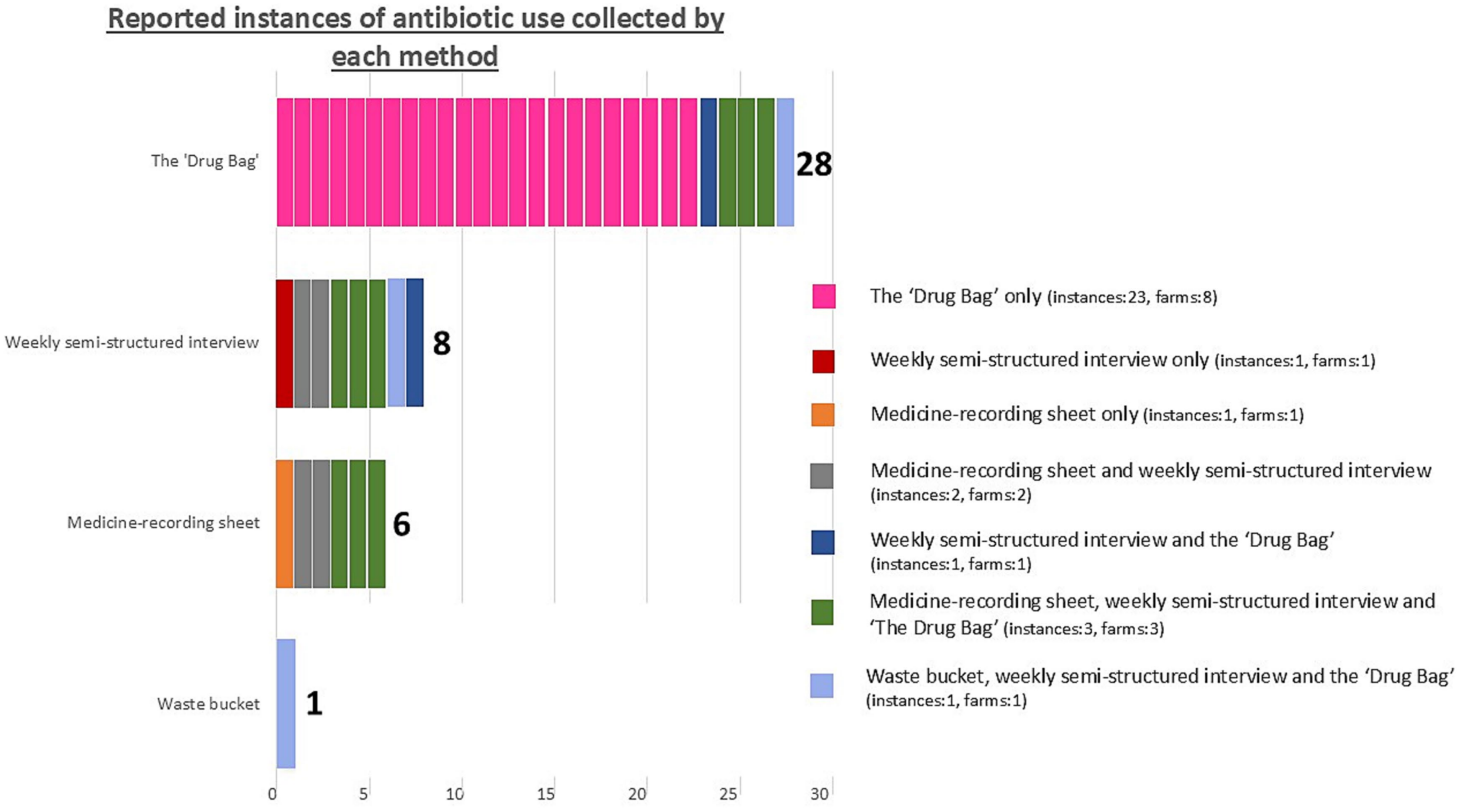
The number of reported instances of antibiotic use identified by each method in this study, evaluating methods to explore antibiotic use on smallholding pig farms in peri-urban Kenya. Colour coding indicates which instances of antibiotic use were captured by one method alone or were triangulated between multiple methods. This is annotated for the number of instances captured by each method and for the number of farms this represents. The total number of instances of antibiotic use we identified during our one-month study period was 32.

**Figure 5:**
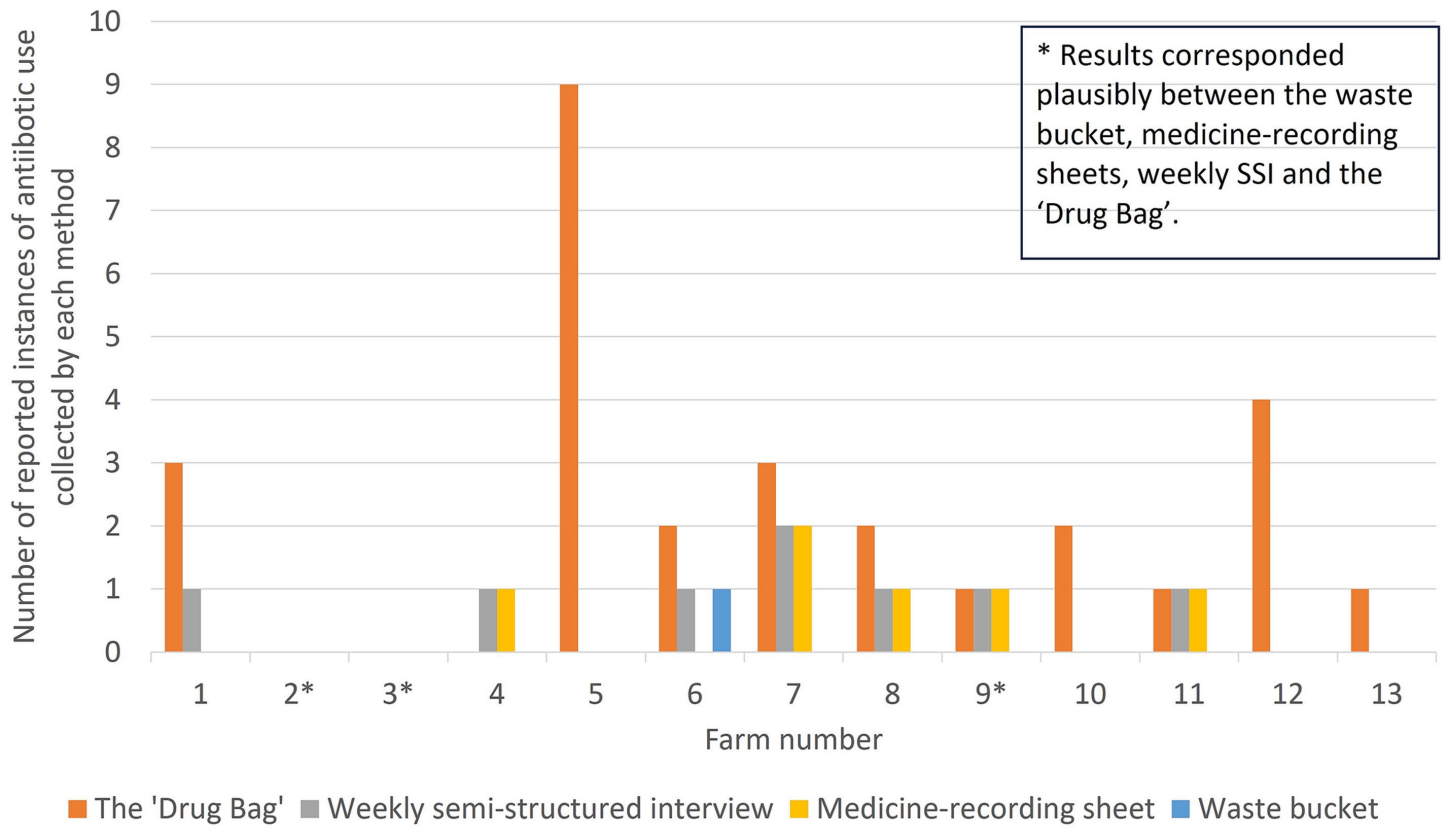
The number of reported instances of antibiotic use identified on each farm and by each method in this study, evaluating methods to explore antibiotic use on smallholding pig farms in peri-urban Kenya. Note that for Farm 11, a different antibiotic was recorded on the medicine-recording sheet than in the “Drug Bag” exercise or through the weekly semi-structured interview, meaning that results did not plausibly correspond between methods.

The number of reported instances of ABU identified by each method is shown in Figure 4, while the number of reported instances of ABU identified for each farm is shown in Figure 5. Without a true measure of ABU on each holding, it is difficult to establish which method captured the most accurate number of instances of ABU. Additionally, by trialing methods together, it is impossible to verify how the use of certain methods influenced the effectiveness of others. That being said, by appraising these data together and triangulating between instances of ABU collected by one method that were not captured by another, several conclusions can be drawn.

Thirty-two injectable or oral ABU instances were reported in total over the study period. No instance of ABU was collected by all four methodsand no single method captured all likely or reported instances of ABU.

Twenty-five out of 32 instances of reported ABU were collected by only one method. This was most common for the “Drug Bag,” for which over-reporting of ABU was most frequent among the four methods. Nine ABU instances were reported by one farmer (Farmer 5) during the “Drug Bag” exercise as being used simultaneously but were not collected through any other method.

Just three out of 13 farms had results that corresponded plausibly between the waste bucket, medicine-recording sheets, weekly SSI, and the “Drug Bag.” Two of these were the only farmers that reported zero antibiotic usage by all four methods (Farmer 2 and Farmer 3). The other farmer (Farmer 9) reported one instance of ABU. This was an injectable antibiotic that did not lead to an empty antibiotic bottle, meaning that, plausibly, the instance of ABU was captured by medicine-recording sheets, weekly SSI, and the “Drug Bag,” but not the waste bucket.

In the next section, we lay out the methodological strengths and limitations identified during the study, organized into seven themes. We provide possible reasons for each method’s under- or over-reporting of instances of ABU and explore the utility of each method for facilitating discussions around ABU practices. Table 2 shows a summary of the strengths and limitations for each method.

**Table 2:** A summary of strengths and limitations for each method.

Method	Number of instances of ABU captured	Strengths for capturing instances of ABU	Limitations for capturing instances of ABU	Success for eliciting ABU practices	General experiences
Waste buckets	1	Over-reporting unlikely.	Under-reported when - AHP administration of antibiotic - Singular doses and part bottles used	Limitations: - Only one ABU instance captured	Strengths: - Easy to use - Collected instances of other medicine use which enriched understanding
Medicine-recording sheets	6	Over-reporting unlikely. Captured three ABU instances by farmers and AHPs that other methods did not.	Under-reported when - Possible fatigue toward the end of the study - Farmers did not record their own ABU - AHP administration of antibiotic	Strengths: - Very detailed explanations of ABU practices when used as a prompt	Strengths: - Participants enjoyed recording - Educated participants on best practice - Collected instances of other medicine use which informed understanding Limitations: - Misunderstanding of clipboard
Weekly semi-structured interview	8	Over-reporting unlikely. Captured one probable ABU instance by an AHP that other methods did not.	If used alone, generally could not definitively ascertain if an antibiotic or another medicine had been given, nor the type of antibiotic.	Strengths: - Trust and rapport were built over weekly visits, leading to openness and frankness in discussions of ABU practices.	Strengths: - Improved understanding of overall healthcare practices - May have improved the accuracy of other methods by reminding participants of the study, minimizing recall bias and building trust Limitations: - Time-consuming
The “Drug Bag” (23)	28	Captured ABU instances that other methods did not, but it is impossible to know how many due to inaccuracies.	Over-reported when - Antibiotics were misidentified as other types of medicines - Time period of 1 month was misremembered - Several antibiotics with the same active ingredient were placed into the same pile Under-reported when - Antibiotics known to have been used were not identified	Strengths: - Inspired interesting discussions around the choice of antibiotics and AHP interactions Limitations: - Memory of ABU practices (e.g., indications for use) often missing, especially when administered by an AHP	Strengths: - Process of collating was useful and insightful for researchers Limitations: - Ethical problems around “showing” participants antibiotics - Expensive and time-consuming to collate - Biosecurity of the bag was challenging

AHP, Animal health professional; ABU, Antibiotic use.

### Strengths of methods used to explore ABU

The strengths we identified for each method evaluated during the study are grouped into three themes: capturing instances of ABU; benefits of methodological plurality; and capturing *more* than instances of ABU.

#### Capturing instances of ABU

Prospective recording methods for ABU (i.e., the waste bucket and medicine-recording sheets) were useful in that they were unlikely to artificially inflate instances of ABU and were not limited by a participant’s ability to remember instances of ABU over a time period. Participants reported that waste buckets were easy to use [see also (18, 20), but unlike (17)]; the volume of total medicines collected (including anti-parasitic medicines or vitamin “boosters”) implied that participants understood the method.

Low literacy levels were not a particular hindrance to medicine-recording sheets in this study context, perhaps due to the peri-urban location of the study. Furthermore, AHPs were the main deliverers of medicines, especially on the farms where literacy may have been a challenge, and were therefore responsible for writing on the medicine-recording sheet.

In some cases, the presence of antibiotic packaging during the “Drug Bag” prompted participants in a way that other methods did not, meaning that reported instances of ABU not detected through other methods were elicited through the “Drug Bag” exercise. During the weekly SSI phase of one interview, a participant described that:

*“During this course of the four weeks you have been coming here, I have not used any drugs.”* (Interview, Farmer 7, November 2022).

However, 15 minutes, during the “Drug Bag” sorting exercise, the same participant said:

*“For example now, those ones, there was one which was, was it this week? Early this week. Used these two* [antibiotics].*”* (Interview, Farmer 7, November 2022).

#### Benefits of methodological plurality

For several instances of ABU captured during the study, one method appeared to support the effectiveness of another. Medicine-recording sheets improved the effectiveness of weekly semi-structured interviews in two cases, where, without the medicine-recording sheet to act as a prompt, participants were unable to recall the name of the antibiotic. Weekly SSIs also seemed to improve the effectiveness of other methods, likely because recall bias is known to become more significant with an increasing length of recall period (39). Visiting farms weekly allowed discussions around reported instances of ABU captured in waste buckets or on medicine-recording sheets to be clear and in-depth, as factors like the clinical signs shown and diagnoses suspected by AHPs were in the recent memory of the participant. Weekly SSIs reminded participants of the study—i.e., that they could use the waste buckets and medicine-recording sheets—which may have improved compliance. The first interim visit was also useful to detect and correct any methodological misunderstandings among participants (explained under a later theme).

#### Capturing *more* than instances of ABU

An understanding of overall healthcare practices on farms is crucial to making evidence-based recommendations on how to reduce inappropriate ABU. The majority (64%) of medicines collected by both prospective methods (waste buckets and medicine-recording sheets) were not antibiotics but multivitamin “boosters,” iron injections, anti-parasitic medicines, one steroid and one vaccine. Rather than this being a hindrance to our study, the collection of non-antibiotic medicines allowed us to better understand pig healthcare practices, such as the role of AHPs on different farms and practices around medicines that farmers appeared more likely to administer themselves, such as anti-parasitic medicines.

Discussing the medicines held on farms during the first visit provided a holding-specific overview of medicine use and preventative healthcare practices. For example, when we asked farmers to describe these preventative healthcare practices, some farmers referred to their use of “vaccines.” Upon further examination of the substances being administered, only one farmer routinely injected pigs with a vaccine as defined by World Health Organization (40) (in this case for swine erysipelas); in other cases, the “vaccines” being administered by farmers were multivitamin injections. While it is possible that this represented a translational issue—another (perhaps more literal) meaning of the Kiswahili word “*chanjo*” is “coverage”— we believe it underlines the complexity and importance of gathering an accurate and contextualized understanding of preventative healthcare practices on farms. As preventative healthcare practices may represent important interventions to improving antibiotic stewardship, misunderstandings or misinterpretations of such practices have the potential to lead to missed opportunities for such interventions.

Weekly SSIs also allowed for rich discussion around pig healthcare on the farms. Over weekly visits, trust and rapport was built, enabling open and frank discussions. Splitting interviews over multiple visits meant that we did not observe participants to show fatigue in the project and weekly visits to farms increased opportunities for observation. This allowed exploration of important themes such as the role of AHPs and medicines on farms. Understanding the challenges of production experienced by farmers allowed our identification of drivers of particular practices – such as ABU (or, more often, drivers for *not* using antibiotics).

Where instances of ABU were captured by medicine-recording sheets, participants were able to provide clear and thorough explanations of ABU practices, using the sheets as prompts. Several participants reported that they liked the concept of recording medicines used on the holding and discussed finding the process to be a learning experience that could equip them with more knowledge going forward. One participant said:

*“It is good to keep records so that you can see if there is one particular disease which is giving you a lot of problems. Then maybe you can even go to your father, even to the university and see what they can help.”* (Interview, Farmer 7, November 2022).

In this way, the exercise also provided helpful insights into the enthusiasm for pig health learning among farmers. Some participants asserted that they would continue to record their medicine use after the end of the project, indicating a positive behavioral change that may be achieved through this type of research.

The “Drug Bag” also elicited useful discussion around participants’ choice of antibiotic products and the role of AHPs on holdings, allowing researchers to begin to uncover some participants’ detailed and complex knowledge of ABU. For example, two participants discussed how they found products labeled with European branding to be most effective. One said:

*“Myself, I do not like Chinese products. They are not so reliable. But drugs from Europe, mostly from Germany, from Sweden, from Netherlands, they are normally very good.”* (Interview, Farmer 7, November 2022).

We found the process of collating the “Drug Bag” useful and insightful as it allowed for further exploration of the study context (see also (23)). During this process, agrovet workers (often veterinarians or veterinary para-professionals themselves) discussed their real-life experiences of antibiotic resistance, including, in one case, their conflict between antibiotic resistance concerns and their need to run a profitable business. This was useful for generating questions and themes to explore through KII and FGD. The process also allowed us to understand the variety of different antibiotics available, including the active ingredients—especially the inclusion of highest-priority critically important antimicrobials (HP-CIAs) (41), indications for use and how the withdrawal period was (variably) displayed on different medicines.

### Limitations of methods used to explore ABU

The limitations identified for each method evaluated during the study could be grouped into four main themes: “*farmers are busy*”—unrealistic expectations of the research project; the context of ABU challenged methodological success; methodological misunderstanding; and project resource considerations.

#### “Farmers are busy”—unrealistic expectations of the research project

Medicine-recording sheets seemed to be most successful in the first 3 weeks of the study, by which point every reported instance of medicine use captured through weekly SSI or waste buckets was also recorded on a medicine-recording sheet. On the final visit, several ABU instances were collected in a waste bucket or discussed through SSI as having taken place in the final week of the study, but were not recorded on the medicine-recording sheet. These examples highlighted the compliance issues that may be introduced by prospective ABU recording methods, which rely on a farmer completing an activity (either placing the antibiotic packaging in the waste bucket or writing the ABU instance on the medicine-recording sheet) at a time when researchers are not present on the farm. Participants’ time was often taken up with other enterprises, responsibilities, and employments, so prioritizing the aims of a research project in all circumstances over several weeks could be an unrealistic expectation.

This potentially unrealistic expectation of participants also appeared relevant to the success of retrospective ABU collection methods (weekly SSI and the “Drug Bag”), which were problematic when participants’ memories of instances of ABU appeared hazy or confused. Although weekly SSI captured one likely instance of ABU not captured by any other methods, in this case the participant could not name the medicine administered as it had been given by an AHP, but it was presumed to be an antibiotic based on the case description. In this way, weekly SSI alone rarely provided details about the specific medicine (e.g., whether it was an antibiotic or another type of medicine) or the diagnosis in cases of clinical disease. Initial SSI also did not elicit any detailed ABU instances. Antibiotics were held on just four of the farms (*n* = 13), and no feed labels that we examined described the inclusion of antibiotics.

Although the “Drug Bag” collated by far the most (28) instances of reported ABU, we assessed this number to be unreliable due to both over- and under-reporting identified in this context. One reason for this discrepancy appeared to be participants being unable to recognize particular antibiotics.

**Figure 6:**
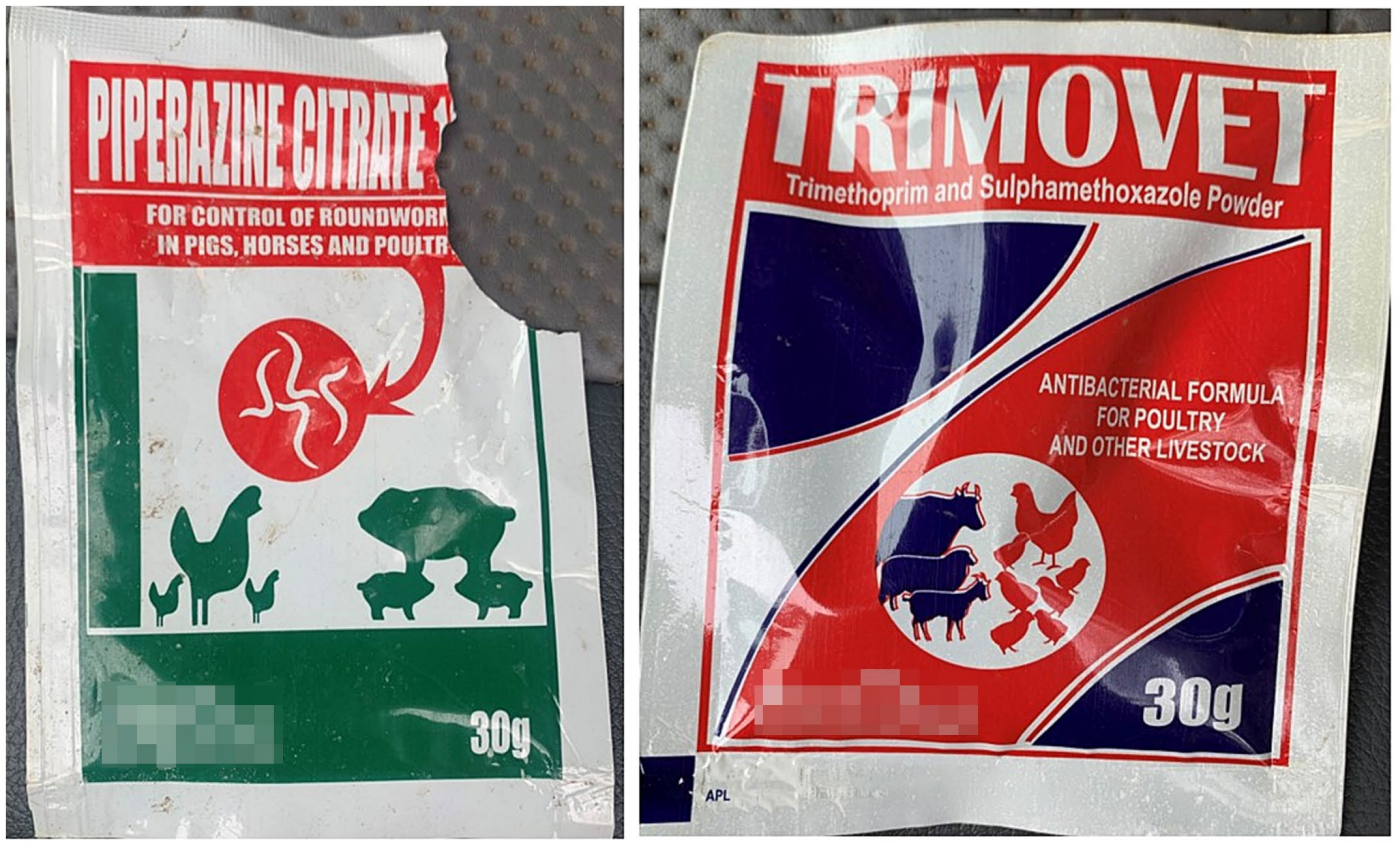
The two medicines that were wrongly identified as the same medicine by Farmer 13 in this study, evaluating methods to explore antibiotic use on smallholding pig farms in peri-urban Kenya. Left is the anti-parasitic medicine the farmer placed in the waste bucket. Right is the antibiotic the farmer identified in the “Drug Bag” sort, saying that this antibiotic was the same as the anti-parasitic medicine they had placed in the bucket.

We documented that at least one participant included an antibiotic in the “used in the last month” pile which was not the medicine that had been used and was instead confused with a visually similar medicine. In this case, during the final week of the study, the participant had placed an oral anti-parasitic medicine in the waste bucket and indicated its use on the medicine-recording sheet for the treatment of “worms” (gastro-intestinal parasites). Approximately 15 min later, the participant placed a similar-looking oral antibiotic powder in the “used in the last month” pile of the “Drug Bag” (see Figure 6). When we asked the participant when the antibiotic in the pile had been used, they answered:

*“This is the one [medicine] that I have given you, in the box. So I give them for the booster, for the itching. I give them for worms.”* (Interview, Farmer 13, November 2022).

This was the only instance where we were able to definitively identify misrecognition leading to over-reporting of ABU for the “Drug Bag” using triangulation of data from other methods. However, we suspected the same in other instances where participants described antibiotics in the “Drug Bag” as multivitamin “boosters,” iron, or anti-parasitic medicines, given that participants consistently described appropriate indications for medicines shown to researchers via the other three methods over the one-month study period. That being said, antibiotics might have also been used by participants for alternative indications during the study.

We also identified two instances where antibiotics were known to have been used during the study duration (as they were identified by other methods) and the exact antibiotic packaging was included in the “Drug Bag,” but participants did not identify the medicine during the “Drug Bag” sort. This meant that the “used in the last month” pile under-reported ABU for these two instances. One of these was an oral powder which had been administered by the participant daily for seven days during the first week of the study. This instance of ABU had been demonstrated during weekly SSI and was also indicated on the medicine-recording sheet.

Several participants could not recall the details around the use of antibiotics which they placed in the “used in the last month” pile. This was especially true when antibiotics had reportedly been administered by an AHP, as is described in the next theme. This made it difficult to ascertain why discrepancies between the “Drug Bag” and other methods were so frequent; this confusion also made it difficult to elicit practices around reported instances of ABU. In one case, a participant separated nine antibiotics into the “used in the last month” pile, which they reported had been injected (all at the same time to the same group of pigs by an AHP) to improve weight gain in pigs shortly before being sold for slaughter. None of these antibiotics had been documented through any other method, and we were unable to ascertain additional details around the report. The plausibility of this account was challenging as it would have constituted a very unusual instance of ABU (two of the products which the farmer reported to have been injected were oral powders; four of these medicines contained a tetracycline antibiotic as an active ingredient, and a further four contained either a penicillin or an amoxicillin). It is possible that the participant was including multiple drugs with similar active ingredients (as we identified elsewhere), that they misrecognized medicines, or that medicines were administered without clinical judgment, possibly by an unregistered AHP. We were unable to ascertain the most likely option and whether this report should be considered genuine. For these reasons, the “Drug Bag” added confusion rather than clarity for five participating farms (*n* = 13), leaving us less confident about participants’ ABU over the previous month.

#### The context of ABU challenged methodological success

At several points in our study, the context in which antibiotics were purchased, prescribed, and administered appeared to challenge methodological success. To explain the lack of ABU instances collected in the waste bucket, participants frequently described that a small number of doses had been administered on an individual animal basis, meaning that an empty bottle or sachet was not available for the bucket.

The role of AHPs as the main deliverers of antibiotics to pigs in this context also appeared to challenge methodological success; instances of ABU which were reportedly administered by an AHP were frequently not captured by one or more methods. For the medicine-recording sheet, given that signage was placed around the farm to ensure knowledge of the project, this demonstrated a lack of compliance from AHPs attending these farms. During the “Drug Bag” exercise, participants described not recognizing antibiotic packaging in the bag because AHPs were employed to prescribe and administer antibiotics on the holding. One farmer said:

*“The vet would know most of them [antibiotics] but us, no. He [the AHP] is the one who comes to treat them [the pigs]. And he knows what to treat.”* [Interview, Farmer 4, November (1)].

In possible explanation of this lack of farmer knowledge of antibiotic products administered by AHPs, both farmers and AHPs (through farm visits, FGDs and KIIs) reported that AHPs may not wish to disclose the identity of injectable medicines so that farmers cannot simply buy that medicine in future without seeking the advice of an AHP beforehand [see (24), for similar findings in Malawi]. One participant said:

*“Farmers are also very tricky. You start telling them the brand names of those drugs and then the next time, they buy it.”* (FGD, Private Veterinarian, November 2022).

Although it is illegal for farmers to administer medicines to farm animals in Kenya (42), this system is rarely enforced and farmers are able to purchase and administer medicines to animals themselves (43, 44).

Another possible explanation for these discrepancies was that AHPs may be nervous about repercussions from detailing ABU practices on the medicine-recording sheets. During FGDs and KIIs, participants often reported their concerns about uncertified AHPs (whom they described as “*quacks*”) illegally attending farms [see (8) for similar findings in Western Kenya]. Such actors may not have trusted the research process and instead viewed it as regulatory.

The level of awareness among pig farmers was unlike that described of poultry farmers in the same country during FGDs [see also (45)]. FGD participants cited this difference in awareness to be for two reasons: firstly, that poultry succumb to more disease than pigs, meaning that ABU is more necessary; and, secondly, due to poultry farmers often receiving sachets which the farmer applies themselves.

We also identified a large variety and fast turnaround of antibiotic products in the study area when purchasing antibiotics for the “Drug Bag,” which may have led to omissions. Many medicines were displayed in similar-looking packaging, which may have contributed to misrecognition of antibiotics during the “Drug Bag” exercise.

#### Methodological misunderstanding

For the medicine-recording sheet, there were two cases where a participant misunderstood the sheet. One participant wrote on the front of the clipboard, rather than inside, and another wrote on the back of the sign. In these cases, the first interim visit was important to correct these issues.

For the “Drug Bag,” at least two participants inflated the “used in the last month” pile by placing all antibiotics with the same active ingredient into this pile, when they described that they had actually only used one example of the drug. This was due to the participants’ extensive knowledge of the active ingredients for each medicine. One participant said,

*“I think we can take all these Pen-Streps [antibiotic]. These ones I think we can say I have used but I do not know now which one.”* (Interview, Farmer 9, November 2022).

Also for the “Drug Bag,” at least two participants wrongly identified the period of one month, meaning that their final “used in the last month” pile over-reported ABU. The initial and final visits were 31 days apart for one participant; however, the participant placed a medicine in the “used in the last month” pile which they then described to have last used before the study began. They said:

*“This month I have only used Kombitrim and Skazone [antibiotics] […] before you came I used this one [Skazone antibiotic] because of diarrhoea for the small ones. Yeah before you came.”* (Interview, Farmer 8, November 2022).

Finally, some participants in the current study expressed that the “Drug Bag” was useful for their education about medicines of which they were not previously aware. This was despite us explaining that the exercise was solely for research purposes and may have inadvertently altered participants’ future ABU. One participant said:

*“We are ok because […] we have seen another medicine that we have not yet used. So we have experience when we get a cow, or a goat, we can get that medicine.”* (Interview, Farmer 10, November 2022).

Similarly to Dixon et al. (23), some participants asked for antibiotics from the “Drug Bag.” We had emptied antibiotic packaging before inclusion of the packaging, meaning that this was less problematic.

#### Project resource considerations

Considerable time was required for travel, completion of weekly SSI, transcription of interviews and in-depth qualitative analysis. Further, the process of buying antibiotics for the “Drug Bag” was time-consuming, expensive and we found maintaining good levels of biosecurity of the bag and its contents challenging in the farm environment.

## Discussion

We evaluated four methods aimed at exploring ABU at the farm level for their ability to capture instances of ABU and elicit ABU practices. We found that no single method captured all likely or reported instances of ABU and that methods were not interchangeable. We identified both under- and over-reporting of ABU for each method, meaning that we were unable to conclude that any one of the methods we trialed to determine farm-level ABU was the most appropriate for this context.

Similarly to Doidge et al. (18), contextual factors influenced the success of ABU collection methods. We encountered accounts that the species of animals kept, the role and attitudes of AHPs and the use of individual (rather than whole group) medication had the potential to impact methodological success. We postulate that the success, and therefore suitability, of methods could be influenced by further contextual factors including farmer education, size of farm, local legislation and enforcement and more.

These findings raise important considerations for researchers completing ABU studies, as well as those seeking to compare farm-level ABU data to compile longitudinal monitoring systems for ABU. These considerations are summarized in our “recommendations to researchers completing ABU studies at the farm level.”

We have demonstrated the need to attain an in-depth understanding of particular study contexts as well as complete pilots of several ABU collection methods before attempting larger ABU studies. The need to understand the study context and complete pilot studies prior to commencing larger monitoring ABU systems is also described by FAO (28) in their recently published guidelines on monitoring ABU. Given that we currently do not have entirely accurate methods for understanding ABU in any context, a perfect method is unlikely to be identified; instead, the use of several methods together may be more successful. This permits cross-checking between ABU collection methods and may mean that similar benefits of methodological plurality experienced in this study could be realized to construct a holistic picture of ABU. Researchers should also consider the need for interim visits, which appeared to minimize recall bias and may have improved the effectiveness of other methods. Gaining the support of AHPs working with farms also has the potential to improve the accuracy of data collected and provide opportunities for greater triangulation.

Reflecting FAO’s recommendation to expand farm-level ABU monitoring in a phased approach (28), our findings show that pilot studies should be extrapolated to, even slightly, different contexts with caution and must be carefully scaled up to study greater numbers of participants; farms across wider geographical areas; or farms over longer time periods. When scaling pilots to longer time periods, more sizeable participant compensation may be required, especially for prospective ABU recording methods where participants must feel motivated to complete exercises when researchers are not present.

When allocating appropriate resources aimed at reducing instances of inappropriate ABU, over-reporting of ABU might be equally as problematic as under-reporting. Therefore, while it might be tempting to utilize the method that collects the most instances of reported ABU in research pilots, our results highlight that doing so may result in the analysis of inaccurate data. To evaluate whether and to what extent chosen methods are likely to under- or over-report ABU for a particular context, we raise the importance of also collecting and analyzing qualitative data around each reported ABU. This could be used to generate estimates of under- or over-reporting for each method, as recommended by Singer et al. (5), and to gather a richer understanding of ABU practices. In this way, in addition to the essential involvement of epidemiologists in farm-level ABU studies, as proposed by FAO (28), our findings suggest the need to also involve social scientists in the planning of such work.

To enable readers of such research to critically evaluate study findings, we suggest that future ABU studies should include a methodological suitability statement which details to what extent the steps and considerations laid out in ‘recommendations to researchers completing ABU studies at the farm level’ (Box 1) have been completed.

BOX 1 **Shows the recommendations to researchers completing ABU studies at the farm level derived from this study, evaluating methods to explore antibiotic use on smallholding pig farms in peri-urban Kenya.**

Recommendations to researchers completing ABU studies at the farm level:

- Conduct pilot studies to understand strengths and limitations of possible methods in the specific study context.
- Generalise findings from pilots or similar studies carefully, by considering how altering the study design or context may affect methodological success.
- Consider using multiple methods in parallel and build in frequent interim visits to farms.
- Collect and analyse qualitative data to examine whether methods are under- or over-reporting ABU and gather a richer understanding of ABU practices.
- Include a methodological suitability statement detailing the extent to which these recommendations have been completed.

## Conclusion

By evaluating four methods to capture ABU for pig farms in Kiambu County, Kenya, we have been able to advance the understanding of methodological approaches used to explore ABU on farms. Our findings support guidelines released by FAO on monitoring ABU at the farm level, which state that pilots should be completed to choose the most appropriate ABU data collection method for a particular context (28). That being said, we raise challenges for those seeking to collect and compare these data.

We were unable to determine one ABU collection method as the most appropriate for this study context. While we found that the use of several methods in parallel with frequent interim visits led to a more thorough understanding of ABU (including ABU practices), the resource intensiveness and expense of such activities may challenge the feasibility of these recommendations for long-term farm-level ABU monitoring systems. For those looking to develop such systems, the complex and intricate strengths and limitations of methodological success that we report suggest that studies must be compared or scaled up to even slightly different contexts (or the same context at a different time) with great care. This is crucial to avoid incomparable results being analyzed together, which could lead to the misinterpretation of interventions or inappropriate allocation of resources.

## Data Availability

The datasets presented in this study can be found in online repositories. The names of the repository/repositories and accession number(s) can be found at: https://reshare.ukdataservice.ac.uk/857083/.
