# Supplementary material for: Evaluating methods to explore antibiotic use on smallholding pig farms in peri-urban Kenya
Source: Front Vet Sci. 2025 Jul 23;12:1570092. doi: 10.3389/fvets.2025.1570092 (PMC12327391; doi:10.3389/fvets.2025.1570092)
Supplement: Supplementary file 2 [file Data_Sheet_2.docx]

**Farmer interview guide:**

This interview schedule is based on ‘AMUSE Livestock- Antimicrobial use in livestock production’ (Weiland et al., 2019). Interviews are semi-structured and participants should be encouraged to talk freely if possible and discuss their thoughts around questions under the same broad focus of heath care practices including antibiotic use on small-scale pig farms in peri-urban Nairobi.

Visit 1

1. Can you show us around the pigs? In doing so, the following questions should be answered.
   1. How many pigs do you have? (Understand numbers of each age/ breeding group)
   2. How do you keep the pigs?
   3. What do you feed the pigs?
   4. What are your biggest challenges to production on the holding?
   5. What do you do to keep the pigs healthy, so that they don’t get sick?
   6. Where do you sell pigs?
   7. What age do you intend on selling pigs to go to slaughter?

Visit 2, 3, 4 and start of Visit 5

1. Discussion of previous week:
   1. Have you experienced any illnesses in your pigs in the last week?
      1. What kind of disease was it?
      2. What action did you take?
      3. Did you turn to anyone for help with diagnosis and treatment? Who?
   2. Have the pigs received any medicines in the last week?
      1. Go to q14
   3. Have you sold any animals to go to slaughter this week?
      1. What age were they?
      2. If not their target age, why did you send them?
      3. Were they given any medicines before selling?
         1. Go to q14
2. Could we go through the waste bucket and medicine record together and discuss how and why each one was used?
   1. Go to q14

Visit 5 continued

1. With the ‘Drug Bag’:
2. Can you sort these into medicines you recognise and those that you don’t?
3. Can you sort this pile (the ‘recognise’ pile) into medicines you have used and medicines you haven’t?
4. Can you sort this pile (the ‘used’ pile) into medicines you use frequently and those you don’t?
5. Can you sort this pile again (the ‘used’ pile) into medicines you have used in the last month?
   1. Go to q14
6. Have you ever experienced a pig dying during or shortly after treatment? Can you describe what you did at this point?
   1. E.g. consume it, bury it, burn it, throw it away
   2. Would you do anything differently if it had been treated?
7. If a pig gets sick shortly before you are supposed to send it to slaughter, what do you do?
   1. What if it receives a medicine?
8. Point to a withdrawal time on a packet of medicine – Do you know what this means?
   1. How often do you follow them?
   2. If sometimes not, why not?
   3. If sometimes not, is there anything that would make you more likely to follow them?
9. Do you have anything that you would like to add?
10. How did you find the study? What bit did you like the most? What bit did you like the least? What about the bin and the chart?
11. Throughout study, for any reported medicines used, try to establish:
    1. Do you know what type of medicine this is / what it does?
    2. How many different times did you use it?
    3. What did you use it for?
    4. Which pigs were given it?
    5. Who administered it?
    6. How was it administered:
       1. For how many days did you give it?
       2. How often did you give it over that time?
       3. How did you give it?
    7. What did you do after giving it? If necessary, probe to understand whether farmers:
       1. Recorded the medicine use
       2. Ate the meat – when?
       3. Sold the pig – when? For what?
       4. If a wait occurred, why did you wait? (Understand if withdrawal time considered.)
    8. Who and where did you get it from?
    9. How did you know how to use it? Did you speak to anyone else about using it?
    10. Did you use any other medicines alongside it?
    11. Was the treatment successful?
    12. Where do you normally keep the medicine?

**Key informant interview guide**

This semi-structured interview is aimed at improving our understanding of how small-scale pig farmers in Kiambu County manage disease and use antibiotics. This is a semi-structured interview - it will be guided by the following questions, although it is anticipated that the discussion will evolve and other topics may be pursued under the same broad focus of disease management and antibiotic use on small-scale pig farms as suggested by the interviewee. The main bullet-pointed questions will be asked and subpoints will only be prompted if not covered by general discussion.

1. Could you introduce yourself and tell us a bit about the nature of your work?
   1. What species do you deal with most?
   2. How often do you speak to small-scale pig farmers, to give them advice about their pigs? How does this compare to how often you speak to larger pig farms?
2. How would you describe the pig farms in your area?
   1. How would you group them?
   2. Which size farms supply to which abattoirs?
   3. Who supplies to small-scale abattoirs?
3. For what reasons does a small-scale pig farmer approach you?
   1. What topics normally arise?
   2. When you are discussing use of medicine with a small-scale pig farmer, what kind of things do you talk to them about?
      1. Do you talk to them about withdrawal periods?
         1. If not, why not?
4. If you are explaining to small-scale pig farmers what a withdrawal period is, what do you say to them?
   1. How important do you think it is that small-scale pig farmers adhere to them?
   2. Do you think it is common for AHSPs to know what a withdrawal time is?
      1. If no / not all AHSPs would know what a withdrawal time is, why do you think that is?
      2. If no / not all AHSPs would know what a withdrawal time is, can you think of anything that would improve awareness?
   3. What proportion of small-scale pig farmers in Kiambu County do you think would know what a withdrawal time is?
      1. If not all, why do you think that is?
      2. If not all, can you think of anything that would improve awareness?
5. How frequently do you think small-scale pig farmers in Kiambu County observe withdrawal times after giving antibiotics?
   1. In what instances do you think they are most likely not to be observed?
   2. Why do you think they may not always be observed?
      1. Which are the most important reasons do you think?
   3. What, if anything, could be done to improve this?
      1. Is there anything that AHSPs could do to improve this?
      2. For example, if you started to mention withdrawal times more frequently when prescribing antibiotics, do you think this would make a difference to how frequently they are observed?
6. Are there any other reasons that you can think of for withdrawal periods not being observed when farmers send pigs to the local independent abattoir?
   1. Explore whether local independent abattoirs could be being used as a salvage option for pigs treated with antibiotics.
7. Is there anything else that you would like to add?

**Focus group discussion guide**

This is a semi-structured focus group - it will be guided by the following questions, although it is anticipated that the discussion will evolve and other topics may be pursued under the same broad focus of antibiotic use practices on small-scale pig farms around Nairobi as suggested by the participants. The main bullet-pointed questions will be asked and subpoints will only be prompted if not covered by general discussion. Throughout the focus group, for the questions that ask for lists of reasons, flip charts and mind maps could be used to track the group’s thoughts.

1. Could you introduce yourself and tell us a bit about the nature of your work? (Go round the group)
2. For what reasons does a small-scale pig farmer approach you for advice?
   1. Rank these in order of how frequently each occurs, (1) being the most frequent.
   2. What topics normally arise?
   3. When you are discussing use of medicine with a small-scale pig farmer, what kind of things do you talk to them about?
      1. Do you talk to them about withdrawal periods?
         1. If not, why not?
3. If, together, you had to explain to small-scale pig farmers what a withdrawal period is, what would you say to them?
   1. Why do you think they exist?
   2. How important do you think they are?
   3. Do you think it is common for AHSPs know what a withdrawal time is?
      1. If no / not all AHSPs would know what a withdrawal time is, why do you think that is?
      2. If no / not all AHSPs would know what a withdrawal time is, can you think of anything that would improve awareness?
   4. What proportion of small-scale pig farmers in Kiambu County do you think would know what a withdrawal time is?
      1. If not all, why do you think that is?
      2. If not all, can you think of anything that would improve awareness?
4. How frequently do you think small-scale pig farmers in Kiambu County observe withdrawal times after giving antibiotics?
   1. In what instances do you think they are most likely not to be observed?
   2. Why do you think they may not always be observed?
      1. Which are the most important reasons do you think?
   3. What, if anything, could be done to improve this?
      1. Is there anything that AHSPs could do to improve this?
      2. For example, if you started to mention withdrawal times more frequently when prescribing antibiotics, do you think this would make a difference to how frequently they are observed?
5. Is there anything else that you would like to add?
