# Supplementary material for: Evaluating methods to explore antibiotic use on smallholding pig farms in peri-urban Kenya
Source: Front Vet Sci. 2025 Jul 23;12:1570092. doi: 10.3389/fvets.2025.1570092 (PMC12327391; doi:10.3389/fvets.2025.1570092)
Supplement: Supplementary file 3 [file Data_Sheet_3.pdf]

Number	Product name	Photo with brand names blurred as belong to third party	Active ingredients	Route of administration
1	S-DIME Bolus<br>Sulfadimidine Bolus	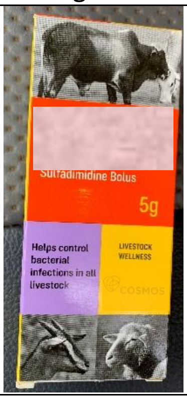 	Sulfadimidine	Oral
2	Skazone<br>TMPS soluble powder	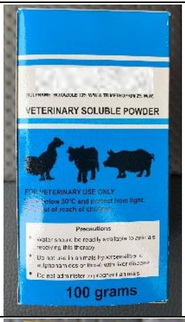 	Sulphamethoxazole<br>Trimethoprim	Oral
3	Egocin 10%<br>Oxytetracycline injection 10%	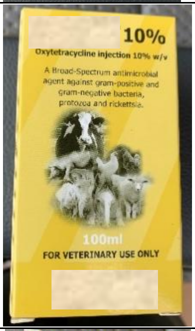 	Oxytetracycline hydrochloride	Injectable
4	Alamycin LA 20<br>Oxytetracycline Injectable Solution	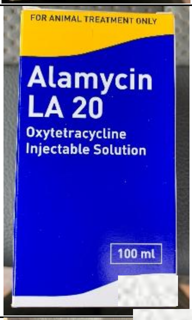 	Oxytetracycline hydrochloride	Injectable
5	Coliveto	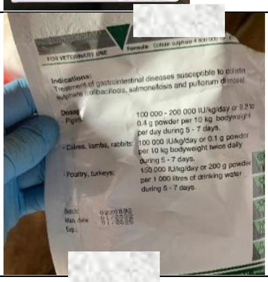	Colistin sulphate	Oral

6	Vetgenta<br>Gentamicin injection 10%	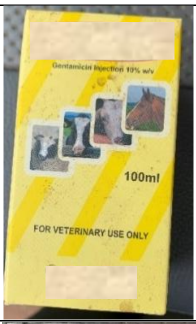	Gentamicin	Injectable
7	Botadoxy<br>Tylosin tartrate and doxycycline hyclate 10%	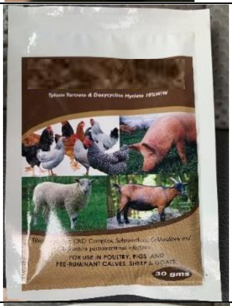	Tylosin Tartrate<br>Doxycycline Hyclate	Oral
8	Trimovet<br>Trimethoprim and sulphamethoxazole powder	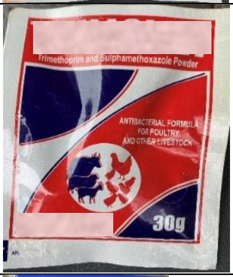	Trimethoprim and sulphamethoxazole	Oral
9	Twiga-penstrep	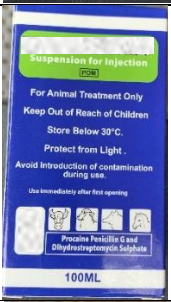	Procaine penicillin G<br>Dihydrostreptomycin sulphate	Injectable
10	Egocin 20% LA<br>Oxytetracycline injection 20%	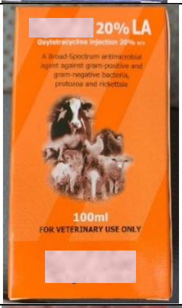	Oxytetracycline hydrochloride	Injectable
11	Bilosin 200 injection	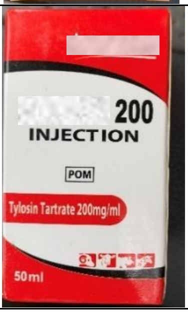	Tylosin tartrate	Injectable

12	Penistrep injection	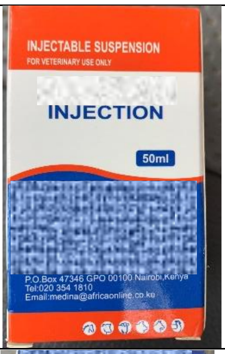	Procaine benzylpenicillin Dihydrostreptomycin	Injectable
13	Damox-LA Amoxicillin 15% Injection	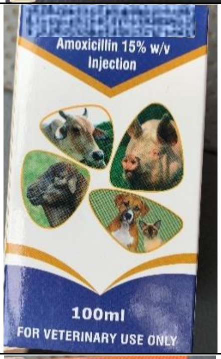	Amoxicillin trihydrate	Injectable
14	Tylosin injection Tylosin tartrate 20%	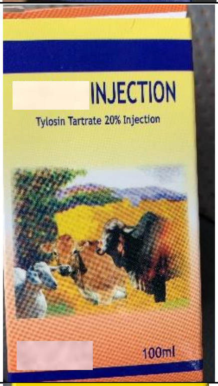	Tylosin Tartrate	Injectable
15	OXY-MET 10 Oxytetracycine 10% injection	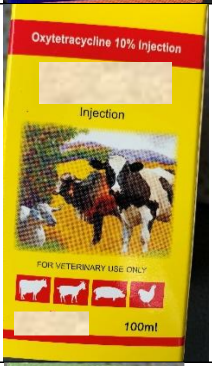	Oxytetracycline hydrochloride	Injectable
16	Dawa Tylosin Tylosin tartrate 200mg/ml 20% injection	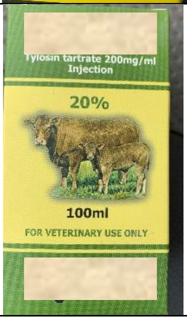	Tylosin Tartrate	Injectable

17	Vetoxy 20<br>Oxytetracycline HCl 20% water soluble powder	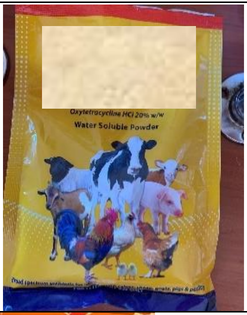	Oxytetracycline hydrochloride	Oral
18	Enrofloxacin Injection 10%	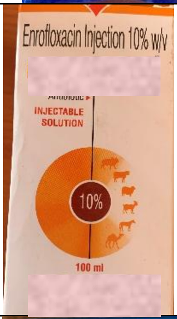	Enrofloxacin	Injectable
19	Alfamox LA<br>Amoxicillin 15% Inj	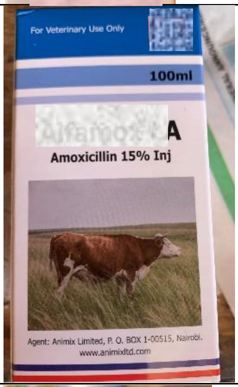	Amoxicillin tihydrate	Injectable
20	Kombitrim 240<br>Sulfamethoxazole + trimethoprim	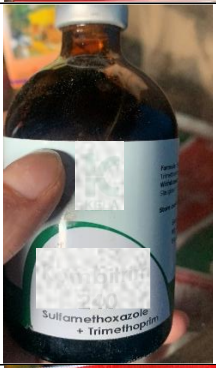	Sulfamethoxazole Trimethoprim	Injectable
21	Tylodoxy 200<br>Tylosin tartrate and doxycycline hyclate Water Soluble Powder	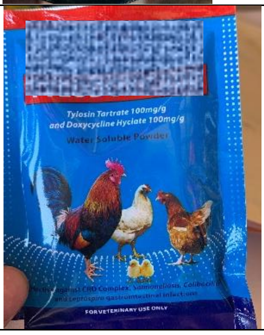	Tylosin tartrate and doxycycline hyclate	Oral

22	Penistrep 20/20	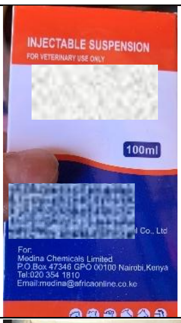	Procaine benzylpenicillin Dihydrostreptomycin	Injectable
23	Tylodoxi 200 Tylosin Tartrate and Doxycycline Hyclate Powerful Antibiotic Formula	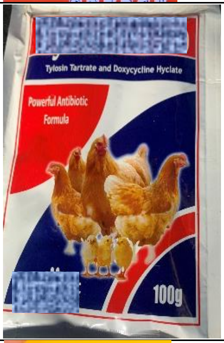	Tylosin tartrate Doxycycline Hyclate	Oral
24	Alamycin LA 300	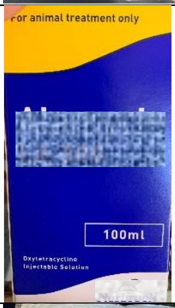	Oxytetracycline hydrochloride	Injectable
25	Agracox	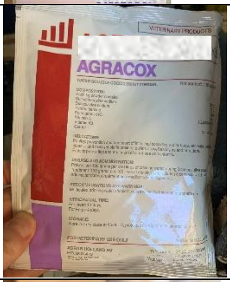	Sulfadimerazine sodium Oxytetracycline	Oral
26	Oxytetra 10%	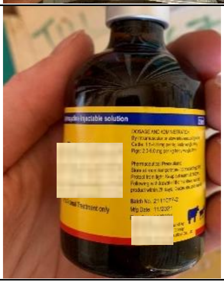	Oxytetracycline hyrochloride	Injectable

27	Aliseryl ws	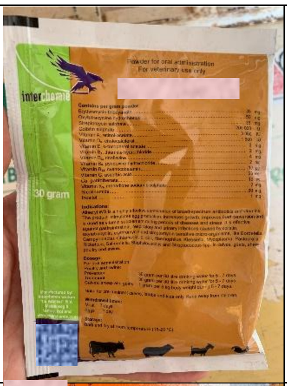	Erythromycin thiocyanate<br>Oxytetracycline hydrochloride<br>Streptomycin sulphate<br>Colistin sulphate	Oral
28	Super skajcycline	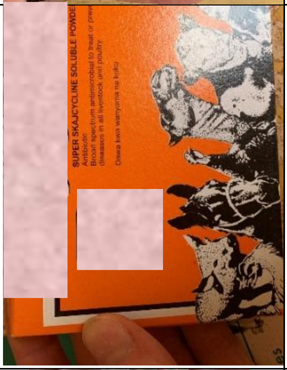	Oxytetracycline hydrochloride	Oral
29	Biocillin-150	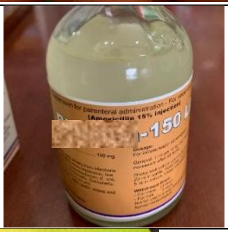	Amoxicillin	Injectable
30	Diseptoprim Bolus Broad Spectrum Antibacterial	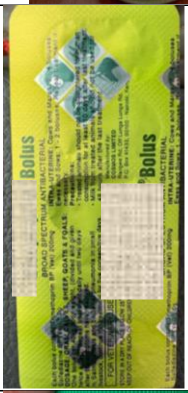	Trimethoprim<br>Sulfadiazine	Oral
31	Oxytet vet-5 WSP	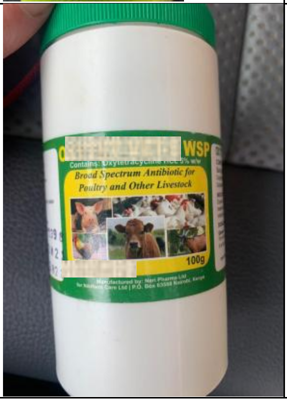	Oxytetracycline HCL	Oral

32	Vetceryl Bactericidal and Bacteriostatic Antibiotic Combination Water Soluble Oral Powder	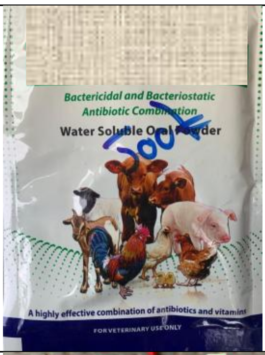	Erythromycin thiocyanate<br>Oxytetracycline hydrochloride<br>Sreptomycin sulphate<br>Colistin sulphate	Oral
33	Dawa Pen Strep	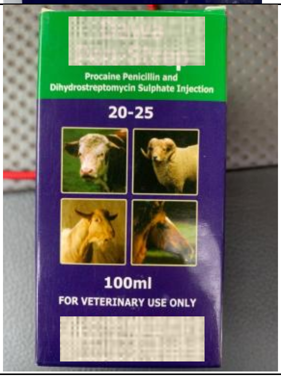	Procaine Penicillin<br>Dihydrostreptomycin Sulphate	Injectable
34	Genta-100<br>Gentamicin	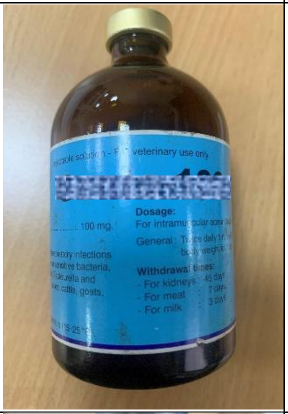	Gentamycin	Injectable
35	Quinitas<br>Enrofloxacin 10%	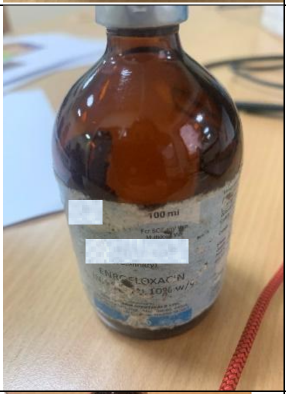	Enrofloxacin	Injectable
36	Hefrotrim	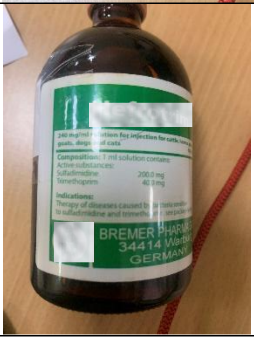	Sulfadimine<br>Trimethoprim	Injectable

37	Super skajcycline	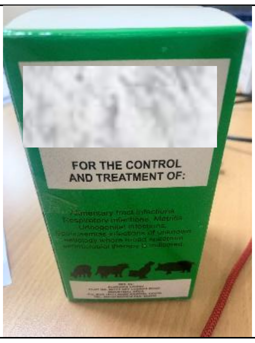	Oxytetracycline hydrochloride	Oral
38	Penstrep – 400	Photo not available	Procaine penicillin G<br>Benzathine penicillin G<br>Dihydrostreptomycin sulphate	Injectable
39	Tetranor 30%	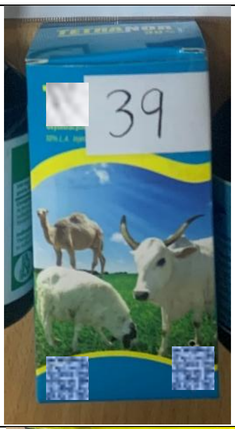	Oxytetracycline hydrochloride	Injectable
40	Biosol Trimethoprim BP 20mg/g and Sulphamethoxazole BP 100mg/g Water Dispersible Powder	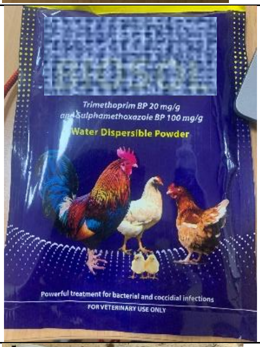	Trimethoprim<br>Sulphamethoxazole	Oral
41	Doxin-200 WS	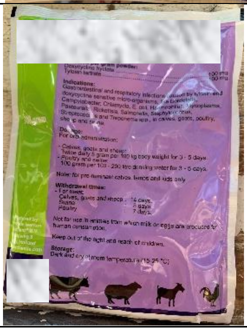	Doxycycline hyclate<br>Tylosin tatrate	Oral
42	Limoxin WS	Photo not available	Oxytetracycline hydrochloride	Oral
